# Sociotechnical Adaptation of Telerehabilitation in Rehabilitation Practice: Survey Among Rehabilitation Professionals

**DOI:** 10.2196/74296

**Published:** 2025-07-28

**Authors:** Tuija Partanen, Riitta Seppänen-Järvelä, Sinikka Hiekkala, Jari Lindh

**Affiliations:** 1 Kela Research, The Social Insurance Institution of Finland, P.O. Box 450, 00056 KELA, Helsinki, Finland, 358 206341944; 2 Faculty of Sport and Health Sciences, University of Jyväskylä, Jyväskylä, Finland; 3 The Finnish Association of People with Physical Disabilities, Helsinki, Finland; 4 Faculty of Social Sciences, University of Lapland, Rovaniemi, Finland

**Keywords:** rehabilitation professionals, sociotechnical adaptation, sociotechnical frameworks, telerehabilitation, outpatient rehabilitation

## Abstract

**Background:**

Both rehabilitation practice and rehabilitation professionals’ work have been transformed by the adoption of different technological solutions. Sociotechnical theory can be used to analyze the adoption of technologies in rehabilitation practice.

**Objective:**

This study aimed to enhance the understanding of the sociotechnical perspective of telerehabilitation (TR) in rehabilitation practice, as well as the understanding of how sociotechnical frameworks can be used to examine the implementation of telerehabilitation in outpatient rehabilitation.

**Methods:**

A survey of rehabilitation professionals (N=629) was conducted in a Finnish outpatient rehabilitation setting. Data from 5 open-ended questions were analyzed using inductive, deductive, and abductive qualitative content analysis. An analysis matrix formed from the Fit between Individuals, Tasks, and Technology (FITT) and the Fit between Individuals, Tasks, Technology, and Environment (FITTE) frameworks was used.

**Results:**

Deductive analysis revealed that in individual-task fit, professionals’ and clients’ adequate skills, support for participation, and a positive attitude were essential. The task-technology fit highlighted the need for the professionals’ familiarization, changes in methods and materials, and collaboration with clients and their close associates and networks. The individual-task fit revealed that professionals found TR tasks to be more complex than those in in-person practice and that TR increased professionals’ perceived workload and clients’ need for personal contact, especially during the familiarization phase. Our findings suggest that certain dimensions of the FITTE framework need to be specified in order to better understand the sociotechnical adaptation of TR.

**Conclusions:**

We propose an extension to create the Fit between Individual, Task, Technology, Interactive Sociotechnical Environment, and Organizational and Sociopolitical Context (FITTIO) framework, which provides conceptual tools for making contextual interpretations of the adoption of TR in rehabilitation settings. This study increases understanding of the sociotechnical nature of TR, which can be used in the adoption of technological solutions in rehabilitation practice.

## Introduction

In the past decades, the adoption of health IT, mobile devices and applications, a variety of web-based platforms and tools, and other digital health technologies has reshaped health care services and professionals’ everyday work [1-3]. There are several different frameworks that can be used to analyze the adoption of technologies in health care, including sociotechnical theory [4]. According to Trist [5], sociotechnical systems theory posits that the main elements of social and technical systems are independent. However, they are also interdependent, as a change in one requires a change in the other. The theory seeks the optimal alignment between these 2 elements [5].

In the context of health care, Ammenwerth et al [6] developed the Fit between Individuals, Tasks, and Technology (FITT) framework. The framework demonstrates the conditions for optimal technology adoption in different health care settings [7-9]. This framework specifically focuses on the intertwined relationships between task, technology, and the individual [6]. The FITT framework has been widely applied and further developed. One research group found that the environment plays an important role in the adoption of technologies [10]. They extended the FITT framework with an environmental dimension, creating the Fit between Individuals, Tasks, Technology, and Environment (FITTE) framework. The environment dimension includes temporal and physical environments as well as organizational policies and procedures [10]. Environmental factors can explain why the adoption of a particular technology is successful in one environment and not functional in another [10-12]. Organizational, management, and leadership factors, as well as clients’ everyday environments, should also be considered within the environmental context [11-14].

At present, research literature concerning the FITT and FITTE frameworks focuses mainly on specific professions such as nurses [6,10,11,14-16] and physicians [10,16-18]. Knowledge of the experiences of case managers, health coaches, social workers, and pharmacists is still quite limited [8,16,17,19]. Some studies have focused on a variety of health care professionals or other workers in health care [11,20-22]. Two studies also included technical staff [9,11]. There is little emphasis on physiotherapy [13] or other therapy professionals [12]. However, the use of telerehabilitation (TR) and factors related to its adoption differ between different professionals [23].

In this paper, we seek to pinpoint the technological and nontechnological factors influencing the adoption of TR in rehabilitation practice. Rehabilitation practice refers to services provided by professional therapists such as music therapists, neuropsychologists, physiotherapists, occupational therapists, and speech and language therapists. It encompasses a wide range of modalities depending on the therapist’s training, the clients’ needs, and the goals of therapy. Therapy can be short-term or long-term, depending on the complexity of the issues being addressed. Here, TR refers to the provision of numerous rehabilitation services through various achievements in information and communication technologies [24].

The goal of this study is twofold: (1) to describe the adaptation of TR in rehabilitation practice from a sociotechnical point of view and (2) to enhance the understanding of how sociotechnical frameworks can be used to examine the adoption of telerehabilitation in outpatient rehabilitation. The present study increases the understanding of the sociotechnical nature of TR, in terms of both technological and nontechnological factors, which can be used in the adoption of technological solutions in rehabilitation practice.

## Methods

### Study Setting

This substudy was conducted in Finland using a web-based survey. The first part of the study investigated and compared the uptake of TR in Finland among different rehabilitation professions during the COVID-19 pandemic [23]. The second part investigated different professional perspectives to provide insight into the extent to which there was consistency in experiences of adopting TR across different professionals [12].

### Data Collection

The focus was on outpatient therapy for intensive medical rehabilitation clients in Finland. The target population comprised professionals who were providing outpatient therapy services in either the public or private sector in Finland during the first wave of the COVID-19 pandemic. A total of 629 professionals, including music therapists, neuropsychologists, physiotherapists, occupational therapists, and speech and language therapists, responded to the questionnaire.

The participants were recruited via email from the Kela directory, networks, and newsgroups of professional organizations, and social media. The questionnaire was anonymous. Responses to all questions were optional. The first page of the web-based questionnaire displayed the informed consent statement. Participants were required to indicate consent to proceed. The web-based survey and the participant recruitment process are illustrated in more detail elsewhere [23].

The questionnaire, designed specifically for this study, consisted of 24 questions regarding rehabilitation professionals’ work and use of TR during the COVID-19 pandemic. Data were collected from May 7 to June 1, 2020. Data for this research consisted of responses to 5 open-ended questions (refer to Multimedia Appendix 1 for the questions used in the study).

### Data Analysis

The study used inductive, deductive, and abductive qualitative content analysis [25-27]. The analysis process is described in Table 1. Initially, the entire dataset was retained and coded using an inductive approach until full saturation was achieved and no new codes were identified. In this process, individual responses or clusters of ideas relevant to the research questions were used as units of analysis. Coding was facilitated using ATLAS.ti Windows (version 22; ATLAS.ti Scientific Software Development GmbH). One author conducted the initial inductive coding, assigning initial codes aligned with the research inquiries. These codes were then subdivided into 2 categories corresponding to the respective research questions, as delineated by Partanen et al [12]. In the deductive and abductive phases, we used an analysis matrix derived from the FITT and FITTE frameworks.

**Table 1.** Description of the analysis process.

Phase of the analysis process	Approach of the analysis	Description of analysis phase
Decontextualization	Inductive approach [25,26]	The first author segmented the texts into meaning units, conducted an initial round of coding, and labeled the codes with descriptive names.
Recontextualization	Inductive approach [25,26]	The first author generated inductive codes through group coding. Inductive codes were discussed among all authors.
Creating categories	Deductive approach [25,26]	The first author sorted the codes into categories. All categories were compared with the dimensions and attributes of the FITT^a^ and FITTE^b^ frameworks. All authors discussed the categories and subcategories, differences in interpretations were discussed, and a consensus was formed. All the dimensions were organized into client-related or professional-related subcategories to provide a more detailed analysis. Codes that could not be included in the analysis matrix led to the creation of additional categories and subcategories.
Abstraction and interpretation of the main categories	Deductive approach [25,26]	Main categories were formulated around the main dimensions of the FITT and FITTE frameworks. Dimensions of fit and environment served as the main categories, with subcategories organized for clients and professionals. The environment dimension was divided into two separate main dimensions: interactive sociotechnical environment and organizational and sociopolitical context*.* All authors reviewed and discussed the categories to ensure consistency in abstraction and interpretation.
Abductive reasoning	Abductive reasoning [27]	A revised framework was created using abductive reasoning by linking the categories and subcategories formed during deductive analysis to the dimensions and attributes of the FITT and FITTE frameworks. Categories that could not be mapped to previous theoretical frameworks were identified during the deductive analysis phase. An abductive leap occurred when the results of the deductive analysis were combined with the dimensions and attributes of the FITT and FITTE frameworks. This abductive reasoning led to a more multidimensional framework, where clients’ and professionals’ perspectives were separated into distinct dimensions, and the concept of the environment was expanded to include two different levels. All authors participated in discussions on abductive reasoning to ensure consistency in definitions and their appropriate application to the data.

a FITT: Fit between Individuals, Tasks, and Technology.

b FITTE: Fit between Individuals, Tasks, Technology, and Environment.

### Ethical Considerations

The present study is part of a larger research project, “The Significance of the Therapeutic Relationship to Rehabilitation” [28]. Ethical approval for the main study, in accordance with the tenets of the World Medical Association Declaration of Helsinki, was obtained from the Ethical Review Board of the Social Insurance Institute of Finland (Kela) in Helsinki (protocol code 4/1/500/2019; November 22, 2019).

## Results

### Overview

The results stemmed from the phased process of inductive, deductive, and abductive content analysis described in the “Data Analysis” section of this paper. We begin by presenting key findings from the deductive analysis (refer to Table 1) that describe the adoption of TR into outpatient rehabilitation practice. The findings from the deductive analysis are presented according to the main dimensions of the FITT and FITTE frameworks. The contents of the main categories are summarized in Multimedia Appendix 2. Subsequently, we present the findings from the abductive analysis, which illustrate how sociotechnical frameworks may be used to examine the implementation of TR in outpatient rehabilitation.

### Individual-Task Fit

We identified 8 main categories in the individual-task fit dimension. The main categories, “Peer support” and “Support for participation,” could not be mapped onto the analysis matrix. Our findings emphasized that an optimal fit between individuals and technology highlighted the importance of skills and abilities, as well as attitudes and preferences. Main categories and subcategories are presented in Multimedia Appendix 2.

Clients need technical skills as well as sufficient motor and cognitive skills. Clients’ challenges in functioning also limited their opportunities to participate in TR. Professionals’ responses primarily focused on the clients’ skills and abilities, with fewer mentions of their skills. However, professionals felt that strong core competence in their profession facilitated the adoption of TR.

The responses pointed out that the suitability of TR is not so much dependent on a client’s diagnosis as it is on personal factors and life situations. Within the category “Support for participation,*”* which emerged from the data, the therapists expressed that in some clients’ situations (for example, clients with severe impairment and pediatric clients), a supportive person makes it possible for the client to participate in TR.

*Physiotherapy for those with severe physical impairments is very difficult to carry out remotely without a supportive person present to help the client.*

[Physiotherapist]

Attitudes and preferences of professionals, clients, and their close associates played an important role in the adoption of TR. Because of negative attitudes, such as prejudice and doubts about technology, the use of TR may not have even been experimented with. The clients did not always have previous experience using remote technology, so experimentation helped them to better understand TR and, thus, assess its suitability for themselves. Professionals sought advice, support, and guidance and also participated in training related to the rapid transition to TR.

### Task-Technology Fit

The dimension task-technology fit included 5 main categories. One main category, “Perceived engagement of clients and their close associates and networks,*”* did not fit the analysis matrix. Main categories and subcategories are presented in Multimedia Appendix 2.

The optimal fit between task and technology included collaboration and the engagement of the client and close associates. These viewpoints emerged in the category, “Perceived Engagement of the Clients and Their Close Associates and Networks.” The professionals reported that tasks given via remote connection require an expanded perspective on engagement. In the subcategory “Remote Connection Supports Network Collaboration and Communication in Rehabilitation Practice,*”* the professionals expressed that communication with other professionals increased, for example, through video conferencing applications. In addition, families, close associates, and other networks were easily reachable.

*On the other hand, these collaborators have been able to take a peek at what I actually do in speech therapy. I think it will affect the cooperation in the future*.

[Speech therapist]

The professionals’ perceptions of the benefits of TR varied. Some of the participants felt that TR, with different forms of connections, activated the client, but others felt that in-person rehabilitation was more useful and effective. The professionals reported changes in practice due to TR. They felt that familiarizing themselves with TR and putting it into practice was time-consuming. Using TR required learning different technologies and guiding clients. TR challenged the professionals’ creativity and competence, as they had to sensibly adapt methods and exercises for remote use, necessitating a re-evaluation of the materials and equipment needed for the practice.

### Individual-Task Fit

We identified three main categories in the individual-task fit dimension. Main categories and subcategories are presented in Multimedia Appendix 2.

The professionals’ responses indicated that preplanning and organizing tasks are more demanding with remote connections, falling into the category “Complexity of task*.*” Professionals found it more challenging to adapt plans on a situation-specific basis compared to in-person meetings, as the client’s environment is either not fully visible or only partially seen.

*Teletherapy requires more precise planning beforehand, and changing the plans when the situation requires it is very challenging when you only have a limited view of the operational environment.*

[Physiotherapist]

The professionals experienced an increased workload with the adoption of TR, both mentally and physically. This type of work increased their cognitive load, requiring significant effort and intense concentration to maintain client interaction via remote connection. In the subcategory of *“*Doing rehabilitation tasks in front of a screen*,”* professionals reported that prolonged use of a tablet or other device could be tiring for clients. In the subcategory “In-person contact during the familiarization phase,” it was considered important for getting to know the client, professionals expressed that getting to know a new person takes time and is easier through in-person contact.

### Interactive Sociotechnical Environment

The interactive sociotechnical environment included 3 main categories. One main category, “Digital environment,” could not be mapped onto our analysis matrix. Main categories and subcategories are presented in Multimedia Appendix 2.

Within the temporal environment, professionals reported that reduced transitions freed up their resources for other purposes. They noted that practicing therapy remotely could save both resources and time. With the shift to TR, adjustments were made to the frequency and duration of rehabilitation appointments as needed. Professionals felt they had the flexibility to schedule sessions according to the clients’ individual needs.

*It’s been easier to book (eg, 2 teletherapy) sessions in the same week when you don’t have to schedule commuting*.

[Occupational Therapist]

*It’s been easier to book (eg, 2 teletherapy) sessions in the same week when you don’t have to schedule commuting*.

[Occupational Therapist]

In the category “Physical environment,” professionals reported learning how to practice therapy remotely from their homes, having a new kind of opportunity to work from home, and having their studies renovated. Remote connection brings a new aspect to the environment that we named “Digital environment.” Remote connection supports the flexibility, accessibility, and regional equality of therapy services. The professionals also felt that the experience of attendance changes with a remote connection. Some participants reported that clients changed appointment times and failed to report cancellations more than in in-person rehabilitation. At the same time, some participants reported that clients’ absences and cancellations decreased because the therapist had a direct connection to the client’s home.

### Organizational and Sociopolitical Context

The organizational and sociopolitical context dimension included 4 main categories. The main category, “Sociopolitical context,” did not fit the analysis matrix. Main categories and subcategories are presented in .

In the “Resources” category, the professionals reported that the transition to TR incurred both one-time and ongoing expenses. In the category “Organization’s goals and processes,” professionals perceived TR’s value as an addition to traditional rehabilitation services. They planned to continue using remote rehabilitation in the future, at least to some extent. In particular, they preferred conducting network and other meetings remotely. Within the subcategory “Information security,” therapists expressed the need for uniform guidelines and practices for the safe use and selection of software. In the category “Current legislation and guidelines,” professionals reported spending a considerable amount of time trying to understand and comply with various instructions and guidelines.

The main category, “Sociopolitical context,” explains how the societal situation affected therapy practice, and professionals had to adopt TR on short notice. The societal situation (ie, the COVID-19 pandemic) forced professionals to take a digital leap.

*The digital leap was enormous, but I’m glad it was taken now that there was no choice. Now there’s no threshold for using telerehabilitation alongside face-to-face rehabilitation*.

[Occupational Therapist]

The professionals reported that this unexpected change caused stress, but on the other hand, it accelerated the adoption of TR. Under normal circumstances, TR would not have been put into practice.

### Extension of the FITTE Framework to the Fit between Individual, Task, Technology, Interactive Sociotechnical Environment, and Organizational and Sociopolitical Context Framework

**Figure 1.**
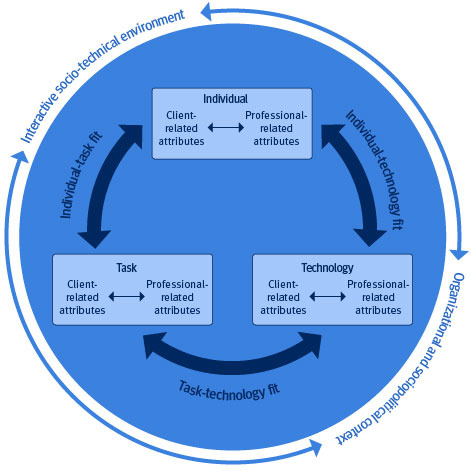
Fit between Individual, Task, Technology, Interactive Sociotechnical Environment, and Organizational and Sociopolitical Context framework.

Abductive reasoning (refer to Table 1) was a creative, dialogic process in which we examined the results of the deductive analysis about the FITT and FITTE frameworks. As a result, the 5 main categories generating refined perspectives on the FITTE framework were identified. In addition, the perspectives of clients and professionals were separated into their dimensions, and the environment was divided into two levels: one describing the interactive, practical work environment, and the other addressing the organizational and sociopolitical context of rehabilitation practice. Based on the abductive reasoning phase, we propose extending the FITTE framework to the Fit between Individual, Task, Technology, Interactive Sociotechnical Environment, and Organizational and Sociopolitical Context (FITTIO) framework. Figure 1 is an extended version of the FITT and FITTE frameworks ([6] and [10], respectively), illustrating the proposed FITTIO dimensions and attributes.

The environmental dimensions, interactive sociotechnical environment, and organizational and sociopolitical context are essential to the FITTIO framework. These dimensions lay the foundation for analyzing and understanding TR practice. The organizational and sociopolitical context defines resources, guidelines, and organizational goals for the use of TR. In this frame, the interactive sociotechnical environment can occur. The interactive sociotechnical environment is the place where professionals meet their clients and collaborate with necessary networks. Both of these environmental dimensions affect all other dimensions.

In FITTIO, the individual-task fit is built on both the clients’ and professionals’ skills, attitudes, and knowledge of how to use a new technical system. From a client’s perspective, the key points are support in participation and professionals’ competence to adjust technological attributes suited for rehabilitation purposes. The optimal task-technology fit focuses on learning how to use the new technical system and the changes it brings to practical work. TR practice emphasizes collaboration with the clients’ networks*.* The individual-task fit dimension focuses on the complexity of the task, mental and physical workload, and personal contact and feedback on performance. The FITTIO framework guides observation through either client-related attributes or profession-related attributes.

## Discussion

### Principal Findings

In TR, the professionals’ practice depends on both social and technical components. Our key findings from the deductive analysis demonstrated that, from the sociotechnical point of view, the adaptation of TR in outpatient rehabilitation practice highlights the fit between individual and technology, as well as the environmental dimensions.

In individual-task fit, adequate skills of professionals and clients, support for participation, and a positive attitude are essential. Our findings align with Kujala et al [11], indicating that most challenges were related to a nonfit between individuals and technology. The professionals in our study expressed concerns that some clients might be unable to use the technology due to disabilities, echoing the findings of Noblin et al [13]. To achieve the optimal task-technology fit, the professionals needed familiarization, changes in methods and materials, and collaboration with the clients and their close associates and networks. The individual-task fit revealed that the professionals found TR tasks to be more complex than in-person practice and that TR increased professionals’ workload and clients’ need for personal contact.

The FITT and FITTE reference questions are particularly well-suited for the design of technology used in clinical settings. When looking at clients in a nonclinical environment, the impact of everyday life, personal context, and environment increases [7,12]. In our study, professionals stated that the sociopolitical situation regarding remote services within the rehabilitation system varied and that there were differences in policies between organizations. Furthermore, in Estel et al [1], most physiotherapists were unaware of the current legal viewpoints related to digitalization. Jacob et al [4] pointed out the importance of organizational aspects in their review of existing theoretical frameworks used in technical adoption. Earlier studies on FITT and FITTE have identified the potential to extend the frameworks to represent these attributes. These studies have reported that policy, management, organizational, interdisciplinary collaboration, and cultural attributes are crucial factors influencing the adoption of TR [10,11,13,14,21].

In our previous study, we pointed out that TR creates a new kind of shared participation [12]. A social system in rehabilitation is traditionally based on the relationships between professionals and clients. According to our findings, social systems in TR cover professional peers, assistive persons, and clients’ close associates’ network engagement. Similar to our findings, Zhai et al [14] suggest extending the FITT framework to cover network engagement and collaboration across the institution. Our findings demonstrate that, to fully understand the sociotechnical adaptation and nonadoption of TR in rehabilitation practice, some of the FITTE framework’s dimensions should be specified more accurately.

### From FITTE to FITTIO

In our abductive reasoning process, we identified refined perspectives and new insights that complement the previous frameworks. Based on these findings, we propose contributing to the evolution of the FITTE framework by introducing the FITTIO framework. FITTIO separates the clients’ and professionals’ perspectives into their dimensions and divides the environmental dimension into two distinct dimensions: an interactive sociotechnical environment and an organizational sociopolitical context*.* This updated framework analyzes the interactions between individual, technology, task, and environmental factors that influence technology adoption in a rehabilitation setting in a more multidimensional manner than previous frameworks.

Similar to Prgomet et al [10], our findings highlight the importance of analyzing the environment as a whole and the involved attributes more precisely. In the original FITT framework, environmental factors were encapsulated as part of individual attributes [6]. Prgomet et al [10] added the environment as its dimension, which helped to analyze the factors related to the context in which individuals, tasks, and technology interplay.

In FITTIO, we apply the proposition of Kujala et al [11] to add context, including organization and management, to the environment dimension, and we divide the dimension into two. The interactive sociotechnical environment describes the interactive, practical environment for therapy. It includes temporal, spatial, and digital qualities, which direct the analysis to when, where, and in what kinds of environments nonfit occurs. The sociopolitical context describes the organizational and sociopolitical attributes that set the frame for the entire rehabilitation practice. With these specifications, FITTIO aims to capture the complexity of the environment of outpatient rehabilitation.

As Ammenwerth et al [6] noted, a detailed analysis using the FITT framework reveals whether problems in the use of technology were based on the nonfit between individual and task, individual and technology, or task and technology. When exploring the adoption of technology from a professional’s perspective, the focus in the framework differs from when examining it from the client’s perspective. Based on our findings, we agree with Kujala et al [11] that both health care professionals and clients should be represented in the individual dimension. Ruyobeza et al [29] suggested that a more detailed task definition may be crucial in assessing the task and skills fit. We agree that a more detailed analysis of required skills, as well as a more precise task analysis, is needed to gain a good understanding of the fit between the individual and the task. In our view, separating the client-related attributes and the professional-related attributes directs the analysis more closely to the needed skills and abilities. Therefore, the FITTIO framework helps to modify tasks, select suitable technology, and guide and help individuals in using the technology or training the skills required.

Frameworks provide shared terminology behind the core conceptual issues, which may be essential for multidisciplinary cooperation in health care and shared interpretation of the problems [4,30]. Sociotechnical systems theories have been used for several decades to explain and understand the interwoven relations of human and technical factors [5]. Since Ammenwerth et al [6] created the FITT framework, it has been used and modified [10,11,29] to gain a better understanding of the adoption of IT systems in health care. When looking at the use of TR as a contextual phenomenon, previous frameworks have not sufficiently addressed the complexities of outpatient rehabilitation. The FITTIO framework provides conceptual tools for making contextual interpretations of the adoption of TR in different clients’ cases in outpatient rehabilitation. With these conceptual tools and shared terminology, the FITTIO framework can strengthen the interdisciplinary engagement in TR development.

The FITTE framework and the sociotechnical theory complement each other in the development of TR. The FITTE framework provides concrete tools and more detailed guidance for analyzing the fit between technology, task, and individual, while sociotechnical theory broadens the perspective toward the FITTIO framework by taking into account the organization, environment, and social context. Therefore, the FITTIO framework can be interpreted as an interconnection of the FITTE framework and sociotechnical theory.

By combining all these viewpoints into the FITTIO framework, the effectiveness and acceptability of TR may be improved, especially when considering both clients’ and professionals’ needs and abilities, as well as the organizational structures and social context. The FITTIO approach ensures that TR services are of high quality and support rehabilitation goals and the implementation of rehabilitation by technology-mediated means.

### Strengths and Limitations

This study has some strengths and limitations. First, the sample included 629 professionals across Finland who were recruited to participate in the survey during the first wave of the COVID-19 pandemic. Due to the anonymous nature of our questionnaire, it was impossible to analyze the differences between professionals who participated and those who did not. Therefore, our study is at risk of nonresponse bias.

It is also important to note that during the first wave of the COVID-19 pandemic, it was strongly recommended to practice rehabilitation only via remote connection. In our sample, many participants conducted TR against their wishes and expressed critical views on TR. The open-ended questions enabled us to identify novel factors related to the sociotechnical adaptation of TR that have not been explicitly studied previously. These insights can be used to further explore optimal design, adaptation, and implementation of TR across different clients and settings.

This study was limited to professionals’ perspectives and lacked insights from clients. Until now, only a few studies using the FITT and FITTE frameworks have focused on clients’ points of view in technology adoption [7,22,31,32]. Thus, future research should include diverse client groups, multidisciplinary providers, and other rehabilitation settings.

In the future, the FITTIO framework should be refined by comparing it with other technology adoption models. The framework still needs to be verified by linking its concepts and their relationships with empirically collected data from different client groups and professionals.

### Conclusion

In TR, professionals’ practice depends on both social and technical components. Based on our findings, which improve the understanding of the FITT and FITTE frameworks in analyzing the technological and nontechnological factors affecting the adoption of TR, we propose an extension from FITTE to FITTIO. In the FITTIO framework, we divided the original dimensions into more specific ones. These specifications allow for a more detailed analysis of TR practice and reveal where adoption and nonadoption occur. In addition, the FITTIO framework provides a shared terminology and conceptual tools for making contextual interpretations of the adoption of TR in outpatient rehabilitation. This study improves understanding of the sociotechnical nature of TR, which may be used in the design and adoption of technological solutions in different rehabilitation settings.

## Supplementary material

10.2196/74296 Multimedia Appendix 1 Open-ended questions used in the study.

10.2196/74296 Multimedia Appendix 2 Main category contents.

## Abbreviations

FITT: Fit between Individuals, Tasks, and Technology
FITTE: Fit between Individuals, Tasks, Technology, and Environment
FITTIO: Fit between Individual, Tasks, Technology, Interactive Sociotechnical Environment, and Organizational and Sociopolitical Context
TR: telerehabilitation
