# Supplementary material for: Sociotechnical Adaptation of Telerehabilitation in Rehabilitation Practice: Survey Among Rehabilitation Professionals
Source: JMIR Rehabil Assist Technol. 2025 Jul 28;12:e74296. doi: 10.2196/74296 (PMC12303547; doi:10.2196/74296)
Supplement: Multimedia Appendix 1 [file rehab-v12-e74296-s001.docx]

Supplementary file 1

Supplementary Textbox 1. Open-ended questions used in the study.

| 1. You can share your thoughts on the impact of the COVID-19 pandemic on your work and telerehabilitation. 2. Have there been changes in the duration and frequency of therapy visits due to telerehabilitation? 3. Have there been changes in the goals of the therapy due to telerehabilitation? 4. With what kind of clients has telerehabilitation not been a possible method of rehabilitation? 5. With what kind of clients have you started implementing telerehabilitation? |
| --- |
