# Supplementary material for: Sociotechnical Adaptation of Telerehabilitation in Rehabilitation Practice: Survey Among Rehabilitation Professionals
Source: JMIR Rehabil Assist Technol. 2025 Jul 28;12:e74296. doi: 10.2196/74296 (PMC12303547; doi:10.2196/74296)
Supplement: Multimedia Appendix 2 [file rehab-v12-e74296-s002.docx]

**Table S1.** Deductive analysis, coding, and individual-technology fit dimension linkage.

| Main category | Professional-related subcategory | Client-related subcategory |
| --- | --- | --- |
| Experience |  | Experimenting with TR^a^ is important |
| Adaptation and adjustment to a new system | Through experimentation, professionals adopted new ways of working and gained positive learning experiences |  |
| Education and training (formal) | Professionals actively sought support |  |
| Peer support^b^ | The importance of peer support was highlighted in the adoption of TR |  |
| IT^c^ knowledge and competencies |  | Participation in TR requires technical skills |
| Skills and abilities | Good core competence in one’s own profession facilitated the adoption of TR | The suitability of TR should be assessed in the rehabilitation plan  Challenges in functionality limit the opportunities to participate in TR independently  The client’s individual skills play a role in participating in TR |
| Attitudes and preferences | Some professionals favored in-person rehabilitation  Professionals’ attitude toward TR changed with the experiment | The attitude of clients and their close associates affected the adoption of TR |
| Support for participation^b^ |  | A supportive person makes it possible for the client to participate in TR |

^a^TR: telerehabilitation.

^b^Categories that could not be mapped onto the analysis matrix.

^c^IT: information technology.

**Table S2.** Deductive analysis coding and task-technology fit dimension linkage.

| Main category | Professional-related subcategory | Client-related subcategory |
| --- | --- | --- |
| Perceived engagement of clients and their close associates and networks^a^ |  | Remote connection supports network collaboration and communication in therapy practice^a^ |
| The system was perceived as useful and valuable | There were differences in the professionals’ experiences of the benefits of TR^b^ |  |
| Familiarization with the functionality of a new technical system | Familiarization with TR and putting it into practice was time-consuming |  |
| Changes in professional practice due to digital systems and devices | Professionals adapted their methods and exercises to be implemented remotely |  |
| Tools, materials, and devices required to perform the task | The use of materials and required equipment had to be re-evaluated |  |

^a^Categories and subcategories could not be mapped onto the analysis matrix.

^b^TR: telerehabilitation.

**Table S3.** Deductive analysis, coding, and individual-task fit dimension linkage.

| Main category | Professional-related subcategory | Client-related subcategory |
| --- | --- | --- |
| Complexity of tasks | Preplanning and organization of tasks to be completed are more demanding with a remote connection than in in-person rehabilitation |  |
| Mental and physical workload | The professionals’ experience of the increased workload was highlighted in the adoption of telerehabilitation | Doing therapy tasks in front of the screen burdened the clients |
| Personal contact and feedback on performance |  | In-person contact during the familiarization phase was considered important for getting to know the client |

**Table S4.** Deductive analysis, coding, and interactive sociotechnical environment dimension linkage.

| Main category | Professional-related subcategory | Client-related subcategory |
| --- | --- | --- |
| Temporal environment | Reduced transitions release the professionals’ resources for other purposes  In the transition to TR^c^, changes were made in the frequency and duration of therapy appointments as needed |  |
| Physical environment | Professionals learned how to practice therapy remotely from their own home |  |
| Digital environment^b^ | Remote connection supports the flexibility, accessibility, and regional equality of rehabilitation services | With a remote connection, the experience of attendance changes, which affects the nonattendance of clients and cancellations of appointments |

^a^TR: telerehabilitation.

^b^Category that could not be mapped onto the analysis matrix.

**Table S5.** Deductive analysis, coding, and organizational and sociopolitical context dimension linkage.

| Main category | Professional-related subcategory | Client-related subcategory |
| --- | --- | --- |
| Resources | During the adoption phase, emphasis was placed on the purchasing of equipment and other financial investments. |  |
| Organization’s goals and processes | TR^c^ was found to be a useful addition to rehabilitation services  Information security. |  |
| Current legislation and guidelines | Differences in the guidelines provided by different organizations affected the work of professionals. |  |
| Sociopolitical context^b^ | The situation in society forced professionals to take a digital leap. |  |

^a^TR: telerehabilitation.

^b^Category that could not be mapped onto the analysis matrix.
